# Metal-Free Organic Chromophores Featuring an Ethynyl-Thienothiophene Linker with an n-Hexyl Chain for Translucent Dye-Sensitized Solar Cells

**DOI:** 10.3390/ma12111741

**Published:** 2019-05-29

**Authors:** Dong-Suk Lim, Kwang-Won Park, Alan A. Wiles, Jongin Hong

**Affiliations:** 1 Department of Chemistry, Chung-Ang University, Seoul 06974, Korea; dsfire89@gmail.com (D.-S.L.); bryan.kwangwon.park@gmail.com (K.-W.P.); 2 WestCHEM, School of Chemistry, University of Glasgow, Glasgow G12 8QQ, UK

**Keywords:** dye-sensitized solar cell, photosensitizer, ethynyl-thienothiophene, computational chemistry

## Abstract

We report the simple synthesis of two organic chromophores featuring an ethynyl-thienothiophene linker with an n-hexyl chain (**CSD-03** and **CSD-04**), their optical and electrochemical properties, and their use as photosensitizers in dye-sensitized solar cells (DSSCs). Our theoretical and experimental studies show that adding the second thienothiophene allows for narrowing the bandgap of the molecule and thus ensuring more light harvesting in the visible region. The efficiencies of both **CSD-03** (5.46 ± 0.03%) and **CSD-04** (5.20 ± 0.03%) are comparable to that of **N719** (5.92 ± 0.01%) in translucent DSSCs fabricated with 5 μm-thick TiO_2_ photoanodes.

## 1. Introduction

The prosperity of a human society is mainly dependent on the supply of sustainable energy. Because of inevitable and imminent shortages of fossil fuels together with global warming, we should look for affordable and clean energy resources. Among renewable energy resources, solar energy is considered a promising way to meet future energy demand. Since the dye-sensitized solar cell (DSSC) was invented in 1991 [1], numerous efforts on materials and device architecture have been made to achieve high performance and long-term stability [2,3,4]. In DSSCs, the chromophore responsible for capturing solar light and producing photocurrent is a critical component to enhance their power conversion efficiency (PCE). For example, Ru-complexes have attained remarkable efficiencies over 11% due to their full absorption window from the visible to the near-infrared (NIR) region [5,6]. However, conventional Ru-complexes have significant drawbacks, such as scarcity, complicated synthesis, and toxicity [2]. Metal-free organic chromophores have been investigated as alternatives because of their many synthetic protocols, high molar extinction coefficients, and reduced toxicity issues [7,8,9]. Among these organic chromophores, a donor-π bridge-acceptor (D-π-A) architecture has been mostly adopted, and a structural modification can tune their photophysical properties to the architecture. Triarylamine-based moieties have been popularly selected as the donor unit because of their expedient synthesis and strong electron donating ability. Cyanoacrylic acid has been extensively used as the acceptor unit due to its direct synthesis from aldehydes and anchoring ability to TiO_2_. Methine or aromatic units have been introduced as π-spacers. However, the selection of an appropriate π-spacer is still of paramount importance in modulating the photophysical properties and enhancing the light-harvesting ability in the visible region. In particular, a planar π-spacer can increase electron delocalization and thus facilitate the electron transfer from the donor to acceptor for higher efficiency.

**Scheme 1:**
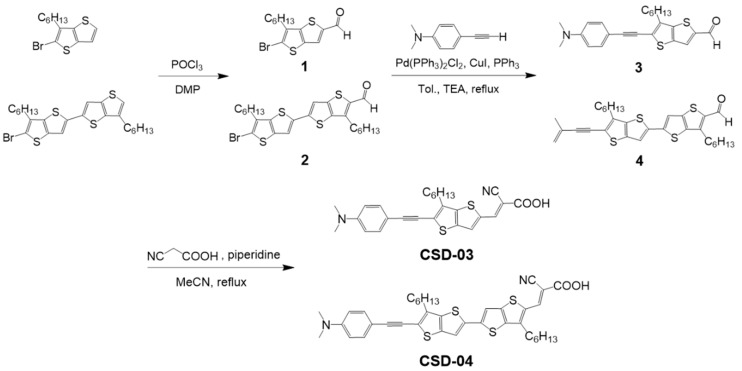
The synthetic route towards dyes **CSD-03** and **CSD-04.**

Thiophene and its derivatives have been widely used as π-bridges because of their low steric hindrance between adjacent molecules and a strong conjugation effect. Also, alkyne units have been used to maintain the planar structure of the D-π-A architecture while increasing the distance between D and A moieties. Recently, ethynyl-thiophene and ethynyl-thienothiophene spacers have been explored theoretically and experimentally. Wang’s group employed thienothiophene and bisthienothiophene linkers to achieve a high molar extinction coefficient of the metal-free organic sensitizers [10]. Recently, we also reported the facile synthesis of two organic dyes featuring ethynyl-thiophene from commercially available starting materials [11,12]. Zhao et al. introduced ethynyl-thiophene or 2-ethynyl-6-methylthieno[3,4-b]thiophene to zinc-porphyrin dyes to enhance the light-harvesting capability and prevent the intermolecular aggregation [13]. Fernandes et al. synthesized three push-pull organic dyes featuring 2,2’-bithiophene, ethynyl-2,2’-bithiophene or ethynyl-thieno[3,2-b]thiophene spacers [14]. In this study, we report two organic dyes, coded as **CSD-03** and **CSD-04** (Scheme 1), which can be synthesized in three steps from commercially available thienothiophene derivatives. The derivative has an n-hexyl side chain in order to improve the solubility of the final compounds. Importantly, the acetylene linker has been included to promote the planarity between the D and A units and thus increase electron delocalization. We also investigate the effect of the number of thienothiophene units on the optical, redox, and photovoltaic properties.

## 2. Results and Discussion

### 2.1. Synthesis

The two compounds, **CSD-03** and **CSD-04**, were directly synthesized in a three-step protocol (Scheme 1). The Vilsmeier–Hack reaction of commercially available thienothiophene derivatives yielded compounds **1** and **2** as the aldehyde. The Sonogashira coupling reactions of compound **1** and **2** with 4-ethynyl-N,N-dimethylbenzenamine provided compounds **3** and **4**, respectively. Subsequently, compounds **3** and **4** were condensed with cyanoacetic acid to obtain **CSD-03** and **CSD-04**, respectively, under Knoevenagel condensation conditions. The two final dyes were fully characterized by ^1^H nuclear magnetic resonance (NMR) spectroscopy, ^13^C NMR spectroscopy, heteronuclear single quantum coherence (HSQC) spectroscopy, Fourier-transform infrared (FTIR) spectroscopy, and high-resolution mass spectrometry (HR-MS) in Figures S1 and S2.

### 2.2. Characterization

**Figure 1:**
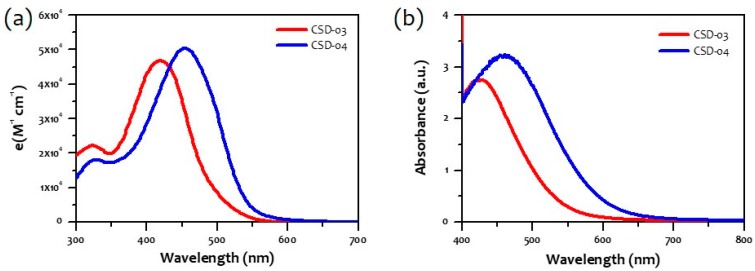
UV-visible absorption spectra of the two dyes (**a**) in DMF solution (1 × 10^−5^ mol L^−1^) and (**b**) adsorbed onto transparent TiO_2_ film.

The UV-visible spectra of the two compounds in dimethylformamide (DMF) and dye-grafted mesoporous TiO_2_ films are shown in Figure 1a,b, respectively. In Figure 1a, two broad absorption bands of each chromophore were observed between 300 nm and 600 nm. The absorption band at lower wavelengths is attributed to localized aromatic π–π* transition, while that at higher wavelengths is ascribed to the intramolecular charge transfer (ICT) from donor to acceptor. The addition of another thienothiophene unit resulted in red-shift in the maximum absorption wavelength (λ_max_) from 419 nm to 455 nm, presumably a result of elongating π-conjugation. The molar extinction coefficients of **CSD-03** and **CSD-04** in DMF were 46,700 M^−1^ cm^−1^ and 50,400 M^−1^ cm^−1^, respectively. Their coefficients are higher than those of the ruthenium-complex dyes N3 (13,900 M^−1^ cm^−1^) [15] and N719 (14,000 M^−1^ cm^−1^) [16]. The optically determined bandgaps were 2.48 eV and 2.25 eV for **CSD-03** and **CSD-04**, respectively. The incorporation of n-hexyl to thienothiophene made the absorption spectra red-shifted and broadened when compared to dye **6** in Reference [14] because of the weaker intermolecular π–π stacking [17]. Compared with the spectra in DMF, the ICT absorption band of the two dyes on TiO_2_ films exhibits significant spectral broadening because of J-aggregation of chromophore molecules and the interaction of the anchoring moieties with the TiO_2_ surface (Figure 1b).

**Figure 2:**
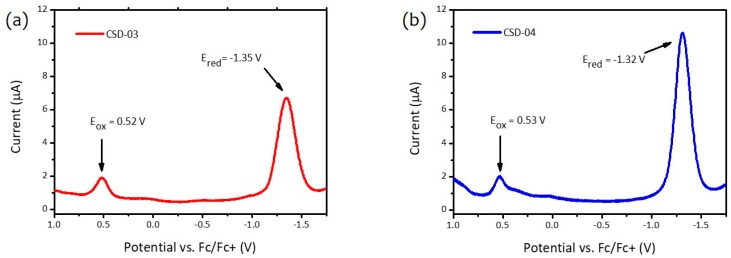
Square wave voltammetry of (**a**) **CSD-03** and (**b**) **CSD-04**.

The electrochemical behavior of the two dyes was investigated by square wave voltammetry (SWV), as shown in Figure 2. Both dyes exhibited a single oxidation wave and a single reduction wave. The addition of the second thienothiophene unit results in more positive reduction potential, ensuring a narrow fundamental energy gap, which correlates to the narrowing of the optical bandgap. According to the energy level of *Fc*/*Fc^+^* redox couple (−4.8 eV under vacuum level [18]), the estimated ionization potentials (IPs) and electron affinities (EAs) are summarized in Table 1. The IPs are slightly lower than the energy level of the *I*^−^/*I*_3_^−^ redox couple (−4.92 eV), confirming that the regeneration of the two dyes is energetically favorable. The EAs are almost 1 eV above the TiO_2_ conduction band (-4.26 eV), indicating efficient electron transfer from the lowest unoccupied molecular orbital (LUMO) of the organic dye to the conduction band of nanocrystalline TiO_2_.

### 2.3. Theoretical Calculations

**Figure 3:**
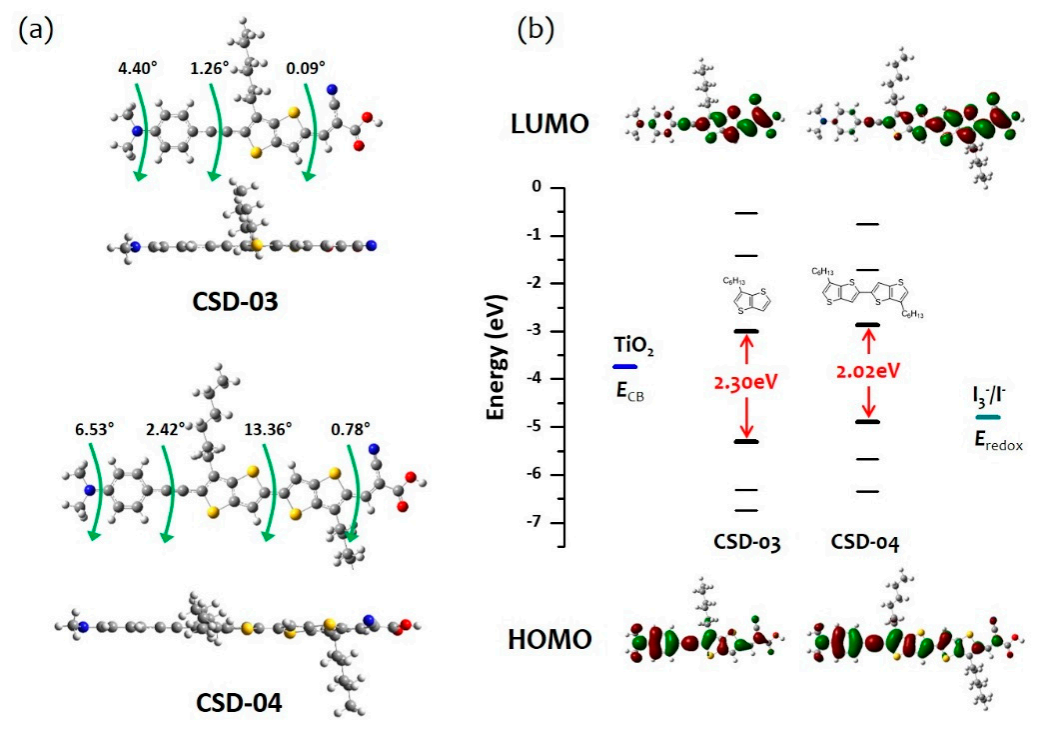
(**a**) Optimized geometries and (**b**) calculated electron distribution and energy levels of **CSD-03** and **CSD-04**.

We performed computational calculations based on density functional theory (DFT) to recognize electron distribution in the frontier molecular orbitals. Figure 3a shows the ground-state optimized geometries of the **CSD-03** and **CSD-04** chromophores. They are fully conjugated throughout the D, π-spacer, and A moieties. The ethynyl-thienothiophene linker allows for maintaining a coplanar molecular structure, which can facilitate the ICT and suppress rotational disorder, even if the n-hexyl chain is on the periphery of the thienothiophene. Interestingly, the ground-state structure of **CSD-04** has a twist angle of 13.4° between two thienothiophene moieties, and thus **CSD-03** has a more planar geometry due to small torsion angles. In Figure 3b, the electrons are distributed from the donor to the π-bridge for both chromophores at the highest occupied molecular orbital (HOMO) level, while the excited electrons are localized on the cyanoacrylic acid at the LUMO level. It is beneficial to the efficient charge transfer from the dye to TiO_2_ upon irradiation. The addition of another thienothiophene unit drives up both HOMO and LUMO levels, in particular, due to a stronger influence on the HOMO. It should be noted that the larger difference between *I*_3_^−^/*I*^−^ potential and the HOMO level allows more efficient dye regeneration because of preventing the geminate recombination between oxidized chromophore molecules and existing electrons in the TiO_2_ under solar illumination.

### 2.4. Dye-Sensitized Solar Cells

**Figure 4:**
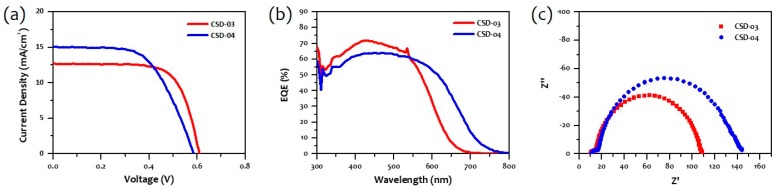
(**a**) Current density-voltage (J–V) characteristics, (**b**) incident photon-to-current efficiency (IPCE) spectra, and (**c**) Nyquist plots in the dark of the masked dye-sensitized solar cells (DSSCs) fabricated with **CSD-03** and **CSD-04**.

Figure 4a displays J-V characteristics of translucent DSSCs (5 μm-thick TiO_2_) sensitized with **CSD-03** and **CSD-04** without any co-adsorbent. The photovoltaic parameters are summarized in Table 2. Adding the second thienothiophene unit resulted in higher *J_sc_* (from 12.55 mA/cm^2^ to 14.85 mA/cm^2^) due to stronger ICT absorption and higher extinction coefficient in the visible region but lower *V_oc_* (from 0.627 V to 0.587 V) due to the up-shift of the HOMO energy level. The photovoltaic properties depending on dye-soaking time were also measured and summarized in Table 2. Interestingly, when compared to **N719**, extended dye-soaking time of **CSD-03** and **CSD-04** resulted in lowering DSSC performance because of the faster aggregation of organic dyes on the TiO_2_ surface. It should be noted that the maximum efficiencies of both **CSD-03** (5.46%) and **CSD-04** (5.20%) are close to that of **N719** (5.92%) in the translucent DSSCs. Figure 4b exhibits incident photon-to-current efficiency (IPCE) spectra of the semitransparent DSSCs. **CSD-04** provides broader IPCE spectrum than **CSD-03**, and thus this agrees with the enhancement of *J_sc_*. However, because of lower *V_oc_* and fill factor (FF), **CSD-04** unexpectedly exhibited lower power-conversion-efficiency (***η***) than CSD-03. FF is mainly affected by many factors, such as the internal resistance in the DSSC relating on the charge transportation at the electrolyte/chromophore/TiO_2_ interface, the charge transfer process at Pt counter electrode and diffusion in the electrolyte and the conductance of transparent electrodes [19]. Also, the dye-containing n-hexyl side chain had better DSSC performance, and IPCE than its counterpart of the nonalkyl chain (dye **6** in Reference [14]) because of its enhanced solubility for dye loading and suppressed π–π stacking. Electrochemical impedance spectroscopy (EIS) can offer a deeper understanding of interfacial charge-transfer processes [20]. Figure 4c shows the Nyquist plots of the DSSCs in the dark. The EIS parameters fitted by an equivalent circuit are summarized in Table 3. The radius of the semicircle in the middle frequency region increases in the order of **CSD-03** (84.46 Ω) < **CSD-04** (111.20 Ω), yielding recombination resistance (*R_rec_*). Large *R_rec_* helps the charge recombination at the electrolyte/chromophore/TiO_2_ interface to be hampered. Unfortunately, **CSD-04** had higher transport resistance (*R_tr_*), shorter electron lifetime (*τ_e_*), and lower charge collection efficiency (*η_cc_*) than **CSD-03** and consequently provided the lower power-conversion-efficiency (***η***) of the DSSC.

## 3. Materials and Methods 

### 3.1. General

^1^H and ^13^C NMR spectra were recorded on a Bruker 500 MHz instrument (Billerica, MA, USA) with tetramethylsilane (TMS) as an internal standard. MS data were obtained using either a JEOL-700 MStation (EI, Akishima, Tokyo, Japan) or a Thermo Scientific LTQ Orbitrap XL (Waltham, MA, USA) with negative ion mode nanoelectrospray ionization (nESI^−^). Infrared (IR) spectra were recorded on a Perkin-Elmer FTIR (Waltham, MA, USA). UV-visible spectra were obtained using a JASCO UV/Vis/NIR spectrophotometer (Tokyo, Japan). Optical bandgaps (*E_g_*) were determined using the edge of the longest wavelength absorption (λ) using *E_g_* = 1240/λ. A three-electrode system consisting of a Pt working electrode, a Pt wire counter electrode, and an Ag wire pseudoreference electrode was used for cyclic voltammetry measurements using a CHI 440A electrochemical analyzer (Austin, TX, USA). The redox potential of the dyes was measured in DMF containing 0.1 M tetrabutylammonium hexafluorophosphate, and the potential was calibrated against the ferrocene/ferrocenium (*Fc*/*Fc*^+^). Before recording the electrochemical data, the solutions were carefully purged with nitrogen gas. 

### 3.2. Syntheses

*Synthesis of 5-bromo-6-hexylthieno[3,2-b]thiophene-2-carbaldehyde (***1***).* 2-bromo-3-hexylthieno [3,2-b]thiophene (1.35 g, 4.44 mmol) and phosphorus oxychloride (2.04 g, 13.4 mmol) were dissolved in DMF (16 mL). The solution was stirred for 30 min at 85 °C and then cooled to room temperature, quenched with H_2_O (20 mL). Dichloromethane (DCM) (200 mL) and H_2_O (200 mL) were added, and the organic layers were separated, dried over MgSO_4_ and filtered. The solvent was evaporated, and the residue was purified by silica gel column chromatography (Hex/EA = 20:1) to give **1** as a dark red oil (0.87 g, 59%). ^1^HNMR (400 MHz, CDCl_3_) δ 0.88 (t, J = 6.78 Hz, 3 H) 1.23–1.45 (m, 4 H) 1.48–1.59 (m, 2 H) 1.71–1.83 (m, 2 H) 3.32 (t, J = 7.53 Hz, 2 H) 8.04 (s, 1 H) 9.56 (s, 1 H) ppm; MS m/z(EI^+^) calculated for C_13_H_15_^81^BrOS_2_ 331.97 found 332.00.

*Synthesis of 5-bromo-6,6′-dihexyl-2,2′-bithieno[3,2-b]thiophene-5′-carbaldehyde (***2***)*. 5-bromo-6,6′- dihexyl-2,2′-bithieno[3,2-b]thiophene (4.24 g, 8.07 mmol) and phosphorus oxychloride (24.7 g, 161 mmol) were dissolved in DMF (40 mL). The solution was stirred for 20 h at 95 °C and then cooled to room temperature, quenched with H_2_O (20 mL). Dichloromethane (MC) (500 mL) and H_2_O (400 mL) were added. The organic layer was separated, dried over MgSO_4_ and filtered. The solvent was evaporated and the residue was purified by silica gel column chromatography (Hex/EA = 10:1) to give **2** as a yellow solid (0.8 g, 18%). ^1^H (400 MHz, CDCl_3_) δ 10.05, 7.32, 7.30, 3.06 (t), 2.75–2.67 (m), 1.79 (q), 1.74–1.66 (m), 1.41–1.29 (m), 0.93–0.86 (m) ppm; MS m/z(EI^+^) calculated for C_25_H_29_^81^BrOS_4_ 554.03 found 554.10.

*Synthesis of 5-((4-(dimethylamino)phenyl)ethynyl)-6-hexylthieno[3,2-b]thiophene-2-carbaldehyde (***3***).* A stirred mixture of 4-ethynyl-N,N-dimethylbenzenamine(0.46 g, 3.2 mmol), **1** (1.00 g, 3.02 mmol), copper(I) iodide (0.012 g, 0.060 mmol), Triphenylphosphine (0.01 6g, 0.060 mmol) and bis(triphenylphosphine)palladium(II) dichloride (0.11 g, 0.039 mmol) in trimethylamine (30 mL) was refluxed for 3 h. After cooling the solution, MC (200 mL) and H_2_O (200 mL) were added. The organic layer was separated, dried over MgSO_4_ and filtered. The solvent was evaporated and the residue was purified by silica gel column chromatography (Hex/EA = 7:1) to give **3** as an orange solid (1.1 g, 92%). ^1^H NMR (400 MHz, CDCl_3_) δ 9.93(s,1H), 7.81(s,1H),6.67(d, J = 8.9 Hz, 2H), 6.61 (d, J = 8.9 Hz, 2H), 3.01 (s, 6H), 2.91 (t, 2H), 1.79 (p, J = 7.6 Hz, 2H), 1.46 – 1.26 (m, 7H), 0.89 (t, J = 7.1 Hz, 3H) ppm; MS m/z(EI^+^) calculated for C_23_H_25_NOS_2_ 395.14 found 394.70.

*Synthesis of 5′-((4-(dimethylamino)phenyl)ethynyl)-6,6′-dihexyl-[2,2′-bithieno[3,2-b]thiophene]-5-carbaldehyde (***4***).* A stirred mixture of 4-ethynyl-N,N-dimethylbenzenamine (0.154 g, 1.06 mmol), **3** (0.56 g, 1.01 mmol), copper(I) iodide (7.7 mg, 0.041 mmol), triphenylphosphine (5.3 mg, 0.020 mmol), bis(triphenylphosphine)palladium(II) dichloride (35.5 mg, 0.0506 mmol) in triethylamine (30 mL) was refluxed for 6 h. After cooling the solution, MC (200 mL) and H_2_O (200 mL) were added. The organic layers were separated, dried over MgSO_4_ and filtered. The solvent was evaporated and the residue was purified by silica gel column chromatography (Hex/EA = 7:1) to give **4** as a yellow solid (0.31 g, 50%).^1^H NMR (400 MHz, DMSO-d_6_) δ 0.83 (t, J = 7.03 Hz, 3 H) 1.23–1.34 (m, 9 H) 2.90 (t, J = 7.28 Hz, 2 H) 2.98 (s, 6 H) 6.74 (m, J = 8.53 Hz, 2 H) 7.39 (m, J = 8.53 Hz, 2 H) 8.28 (s, 1 H) 8.57 (s, 1 H) ppm; MS m/z(EI^+^) calculated for C_35_H_39_NOS_4_ 617.96 found 617.50.

*Synthesis of (E)-2-cyano-3-(5-((4-(dimethylamino)phenyl)ethynyl)-6-hexylthieno[3,2-b]thiophen-2-yl) acrylic acid (**CSD-03**).* A mixture of **2** (1.1 g, 2.8 mmol) and cyanoacetic acid (0.50 g, 5.6 mmol) was dissolved in CH_3_CN (50 mL) containing piperidine (1.42 g, 16.7 mmol). The solution was refluxed for 3 h. After cooling the solution, it was poured into HCl (2 M, 200 mL) and MC (500 mL). The organic layer was separated, washed with water, dried over MgSO_4_, and filtered. Then the solvent was removed in vacuo to afford **CSD-03** as a dark red solid (0.3 g, 23%). ^1^H NMR (500 MHz, DMSO-d_6_) δ 8.47 (s, 1H), 8.20 (s, 1H), 7.39–7.37 (m, 2H), 6.74–6.72 (m, 2H), 2.97 (s, 6H), 2.89 (t, J = 7.4 Hz, 2H), 1.76–1.70 (m, 2H), 1.34 – 1.22 (m, 6H), 0.83 (t, J = 7.2 Hz, 3H) ppm.; ^13^C(126 MHz, DMSO-d_6_) δ 163.8, 151.0, 145.9, 138.7, 138.0, 137,2, 132.9, 131.8, 117.6, 112.3, 107.6, 101.7, 100.0, 81.0, 40.1, 31.2, 28.6, 28.5, 28.2, 22.4, 14.4 ppm; FT-IR (ATR) υ 2920, 2852, 2181, 1672, 1603, 1568, 1533, 1470, 1418, 1368, 13.12, 1244, 1192, 1148, 1125, 945, 832, 812, 758, 745, 608, 584 cm^−1^; HRMS m/z (FAB^+^) calculated for C_26_H_26_N_2_O_2_S_2_ [M]^+^ 462.1436, found 462.1439.

*Synthesis of (E)-2-cyano-3-(5-((4-(dimethylamino)phenyl)ethynyl)-6,6′-dihexyl-2,2′-bithieno[3,2-b] thiophen-5′-yl)acrylic acid (**CSD-04**).* A mixture of **4** and cyanoacetic acid (0.0173 g, 0.204 mmol) was dissolved in CH_3_CN (40 mL) containing piperidine (0.052 g, 0.061 mmol). The solution was refluxed for 5 h. After cooling the solution, it was poured into HCl (2 M, 200 mL) and MC (500 mL). The organic layer was separated and washed with water, dried over MgSO_4_, and filtered. Then, the solvent was evaporated in vacuo to afford **CSD-04** as a red solid (0.056 g, 80%). ^1^H NMR (500 MHz, DMSO-d_6_) δ 8.22 (s, 1H), 7.79 (s, 1H), 7.76 (s, 1H), 7.45 (d, J = 9.0 Hz, 2H), 7.01 (s, 1H), 6.72 (d, J = 9.0 Hz, 2H), 2.96 (s, 6H), 2.89–2.86 (m, 4H), 1.69–1.63 (m, 12H), 0.86–0.83 (m, 6H) ppm.; ^13^C(151 MHz, DMSO-d_6_) δ 168.6, 150.4, 145.5, 141.8, 141.7, 140.6,139.5, 138.6, 138.3, 137.9, 136.9, 134.7, 133.0, 132.2, 125.6, 121.3, 119.2, 118.1, 117.0, 112.0, 104.6, 40.1, 31.0, 30.9, 29.1, 28.8, 28.4, 28.4, 27.9, 27.5, 22.0, 21.6, 14.0, 13.9, 13.9 ppm; FT-IR (ATR) υ 2911, 2854, 2202, 1763, 1603, 1521, 1458, 1427, 1363, 1312, 1283, 1204, 1182, 1105, 1057, 945, 810 cm^−1^; HRMS m/z (FAB^+^) calculated for C_38_H_41_N_2_O_2_S_4_ [M+H]^+^ 685.2051, found 685.0078.

### 3.3. Computational Details

Theoretical calculations were performed with the Gaussian 09 [21]. The ground-state geometries were fully optimized using the DFT method with Becke’s three-parameter hybrid exchange functionals and the Lee-Yang-Parr correlation functional (B3LYP) and 6-311G (d, p) basis set for all atoms without additional diffused function. Vibrational frequencies were also calculated to confirm that the optimized geometry would correspond to the lowest point of the respective potential energy surface.

### 3.4. DSSC Fabrication and Photovoltaic Measurements

The acetylene-black TiO_2_ paste [22] was screen-printed onto fluorine-doped SnO_2_ (FTO) conducting glass (TEC 8, Pilkington, 2.2-mm-thick, sheet resistance = 8 Ω/sq). The printed films were placed in a muffle furnace and slowly heated to 300 °C over a 30 min period, heated at 300 °C for 1 h, heated to 575 °C for 1 h, and then cooled to room temperature after 3 h. The final thickness of the sintered films was about 5 μm, and their active area was 0.2025 cm^2^ (i.e., 0.45 cm×0.45 cm). The prepared TiO_2_ photoanodes were dipped in a 0.04 M TiCl_4_ solution at 75 °C for 30 min, rinsed several times with deionized water and ethanol, and then annealed at 500 °C for 30 min on a hot plate. The photoanodes were treated with the O_2_ plasma for 10 min, and then immersed in 0.1 M HNO_3_ solution for 30 min to facilitate dye adsorption. The final ones were submerged in a 0.5 mM dye-containing ethanol solution (**N719**, **CSD-03**, and **CSD-04**) for different soaking time. After two holes were drilled in the FTO-coated glass, chloroplatinic acid (H_2_PtCl_6_) solution in 2-propanol was spin-coated and annealed at 525 ºC for 1 h in the muffle furnace to prepare the Pt counter electrode. Both the dye-grafted photoanode and the counter electrode were assembled with 25-μm-thick Surlyn (Solaronix, Aubonne, Switzerland). A liquid electrolyte (Iodolyte AN-50, Solaronix) was injected through the holes in the backside of the counter electrode.

The photovoltaic performance of the masked devices was investigated by using a solar cell I-V measurement system (K3000 LAB, McScience, Suwon, Korea) under the standard AM 1.5G spectrum (100 mW cm^−2^). Short-circuit photocurrent density (*J*_sc_), open-circuit voltage (*V*_oc_), fill factor (*FF*), and power conversion efficiency (*η*) were obtained simultaneously. The rectangular mask size (e.g., 0.6 cm × 0.6 cm) was somewhat larger than the active area of the photoanodes because this is the preferred configuration to capture both direct and diffuse lights and to minimize internal reflection [23]. The incident photon-to-current conversion efficiency (IPCE) was obtained using a spectra IPCE measurement system (K3100, McScience, Korea). EIS experiments were performed with cells biased to *V*_oc_ in the dark using a frequency response analyzer (Solartron 1260, AMETEK, Leicester, UK). A sinusoidal potential modulation of 10 mV was applied over a frequency range from 0.1 Hz to 100 kHz. The obtained EIS spectra were fitted using the equivalent circuit built in the ZView software (Version 3.5f, Scribner Associates Inc., Southern Pines, NC, USA).

## 4. Conclusions

In this article, we report the facile synthesis of the two organic dyes featuring an ethynyl–thienothiophene linker with an n-hexyl chain, which will add impetus to develop lower cost photosensitizers for the DSSCs. UV-vis spectroscopy and square wave voltammetry have shown that adding the second thienothiophene allowed for narrowing the bandgap of the molecule and ensuring better light harvesting in the DSSCs. Unexpectedly, the extended π-conjugation negatively affects the structural planarity and HOMO level of **CSD-04**. Therefore, the photovoltaic performance of **CSD-03** (5.45 ± 0.03%) is better than that of **CSD-04** (5.07 ± 0.18%).

## Figures and Tables

**Table 1:** Summary of optical and electrochemical properties of the as-synthesized dyes.

Dyes	*λ_max_*/*λ_onset_* (nm)	*E_g_ (optical)* (eV)	*E_ox_* (V)	*E_red_* (V)	*IP* (eV)	*EA* (eV)	*E_g_ (e-chem)* (eV)
**CSD-03**	419/499	2.48	0.52	−1.35	−5.32	−3.45	1.87
**CSD-04**	455/550	2.25	0.53	−1.32	−5.34	−3.48	1.85

**Table 2:** Masked DSSC (5 μm-thick TiO_2_) performance parameters of **CSD-03**, **CSD-04**, and **N719**.

Dyes (Soaking Time)	*V_oc_* (V)	*J_sc_* (mA/cm^2^)	*FF*	*η* (%)
**CSD-03** (3 h)	0.627 ± 0.013	12.55 ± 0.18	69.4 ± 1.1	5.46 ± 0.03
**CSD-03** (6 h)	0.619 ± 0.022	12.64 ± 0.68	69.1 ± 0.2	5.41 ± 0.08
**CSD-03** (12 h)	0.6111 ± 0.004	11.15 ± 0.51	68.7 ± 0.3	4.69 ± 0.27
**CSD-04** (3 h)	0.587 ± 0.004	14.85 ± 0.29	58.2 ± 1.4	5.07 ± 0.18
**CSD-04** (6 h)	0.576 ± 0.009	13.86 ± 0.35	65.2 ± 1.0	5.20 ± 0.03
**CSD-04** (12 h)	0.587 ± 0.016	12.974 ± 0.42	64.1 ± 0.9	4.88 ± 0.01
**N719** (3 h)	0.608 ± 0.003	14.74 ± 0.47	62.1 ± 0.1	5.57 ± 0.21
**N719** (24 h)	0.671 ± 0.001	14.95 ± 0.39	59.1 ± 0.6	5.92 ± 0.01

**Table 3:** Electrochemical impedance spectroscopy (EIS) fitting parameters of the DSSCs (5 μm-thick TiO_2_).

Dyes	*R_tr_* (Ω)	*R_rec_* (Ω)	*C_μ_* (mF)	*τ_e_* (ms)	*η_cc_* (%)
**CSD-03** (3 h)	5.68	84.46	0.08	6.34	93.70
**CSD-04** (3 h)	11.99	111.20	0.05	5.65	90.27

## Supplementary Materials

The following are available online at https://www.mdpi.com/1996-1944/12/11/1741/s1, Figure S1: (a) FTIR, (b) ^1^H NMR, (c) ^13^C NMR, and (d) HSQC spectra of CSD-03, Figure S2: (a) FTIR, (b) ^1^H NMR, (c) ^13^C NMR, and (d) HSQC spectra of CSD-04.
