# Supplementary material for: Metal-Free Organic Chromophores Featuring an Ethynyl-Thienothiophene Linker with an n-Hexyl Chain for Translucent Dye-Sensitized Solar Cells
Source: Materials (Basel). 2019 May 29;12(11):1741. doi: 10.3390/ma12111741 (PMC6600718; doi:10.3390/ma12111741)
Supplement: Supplementary file 1 [file materials-12-01741-s001.pdf]

materials

MDPI

Supplementary Materials

Metal-Free Organic Chromophores Featuring an Ethynyl-Thienothiophene Linker with an n-Hexyl Chain for Translucent Dye-Sensitized Solar Cells

Dong-Suk Lim [1,†], Kwang-Won Park [1,†], Alan A. Wiles [2,*] and Jongin Hong [1,*]

1 Department of Chemistry, Chung-Ang University, Seoul 06974, Korea; dsfire89@gmail.com (D.-S.L.); bryan.kwangwon.park@gmail.com (K.-W.P.)

2 WestCHEM, School of Chemistry, University of Glasgow, Glasgow G12 8QQ, UK

* Correspondence: Alan.Wiles@glasgow.ac.uk (A.A.W.); hongj@cau.ac.kr (J.H.); Tel.: +441413302037 (A.A.W.); +8228205869 (J.H.)

† These authors contributed equally to this work.

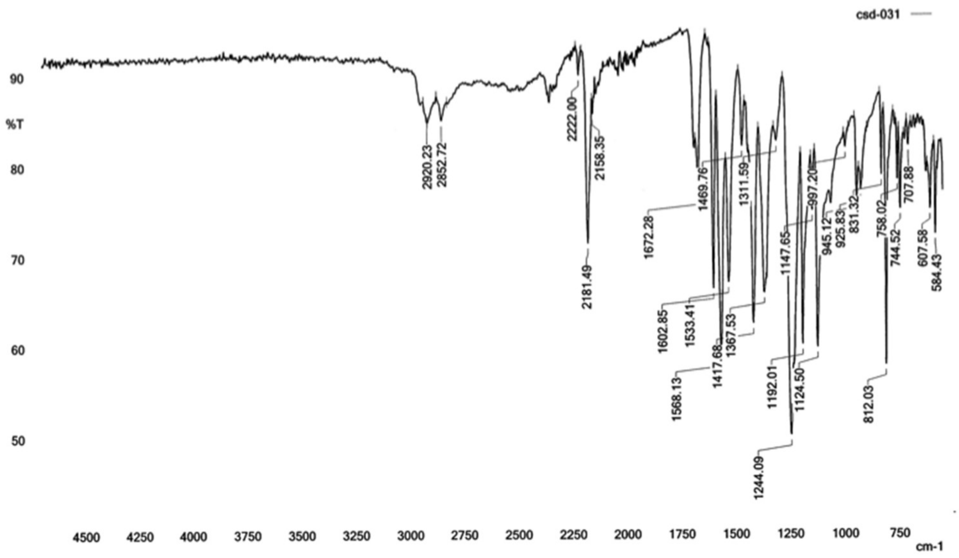

(a)

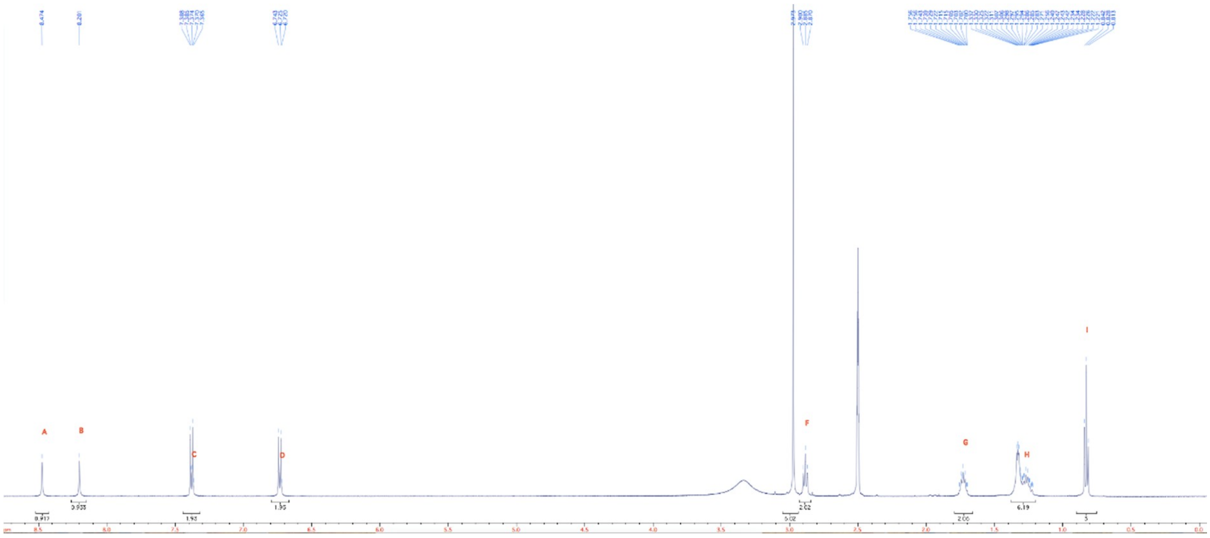

(b)

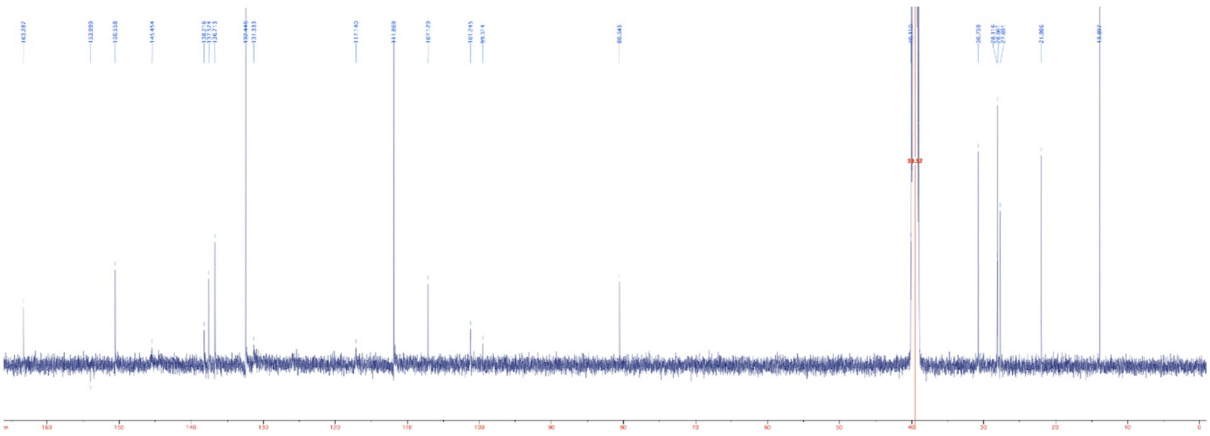

(c)

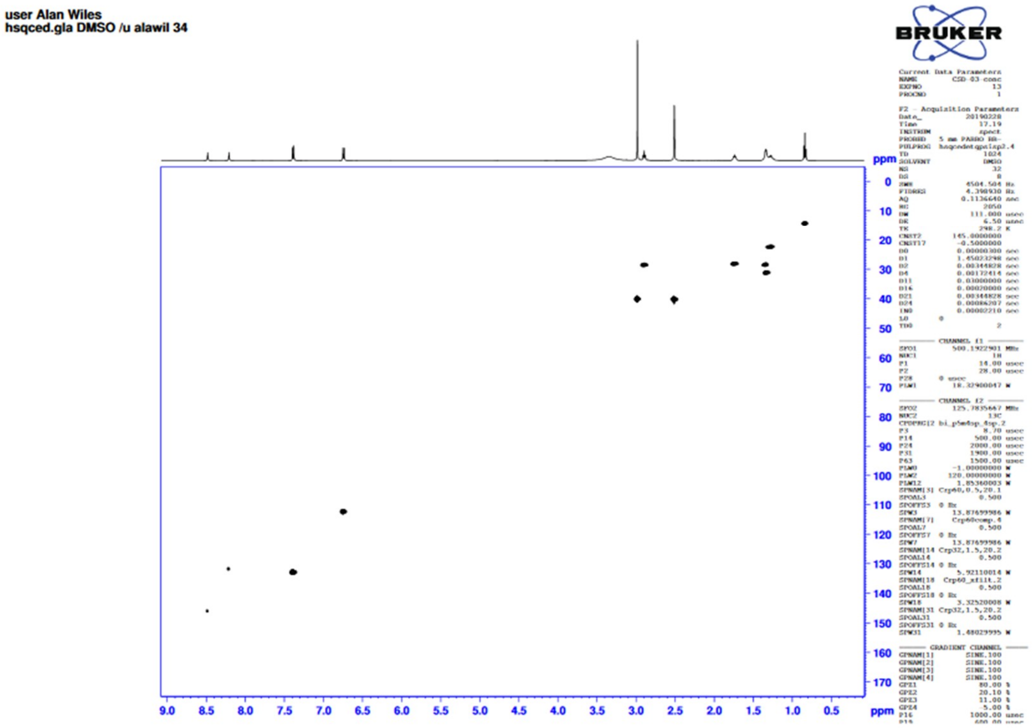

(d)

Figure S1. (a) FTIR, (b) 1H NMR, (c) ^{13}C NMR, and (d) HSQC spectra of **CSD-03.**

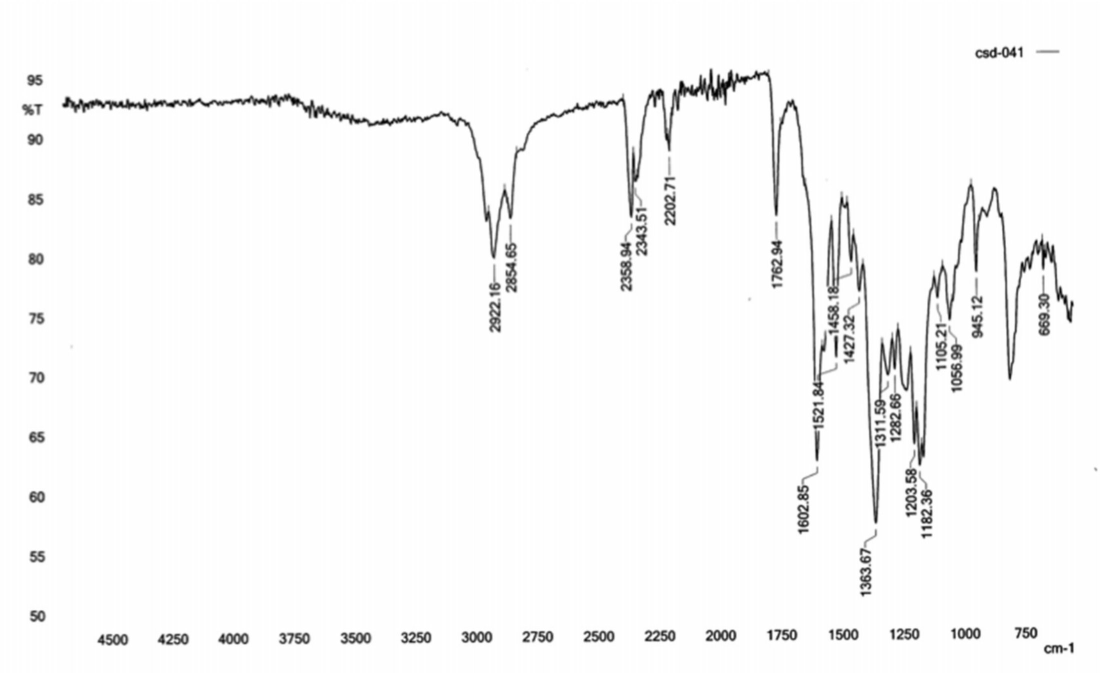
csd-041
%T
95
90
85
80
75
70
65
60
55
50
2922.16
2854.65
2358.94
2343.51
2202.71
1762.94
1458.18
1427.32
1521.84
1602.85
1311.59
1282.66
1363.67
1203.58
1182.36
1105.21
1056.99
945.12
669.30
4500 4250 4000 3750 3500 3250 3000 2750 2500 2250 2000 1750 1500 1250 1000 750
cm-1

(a)

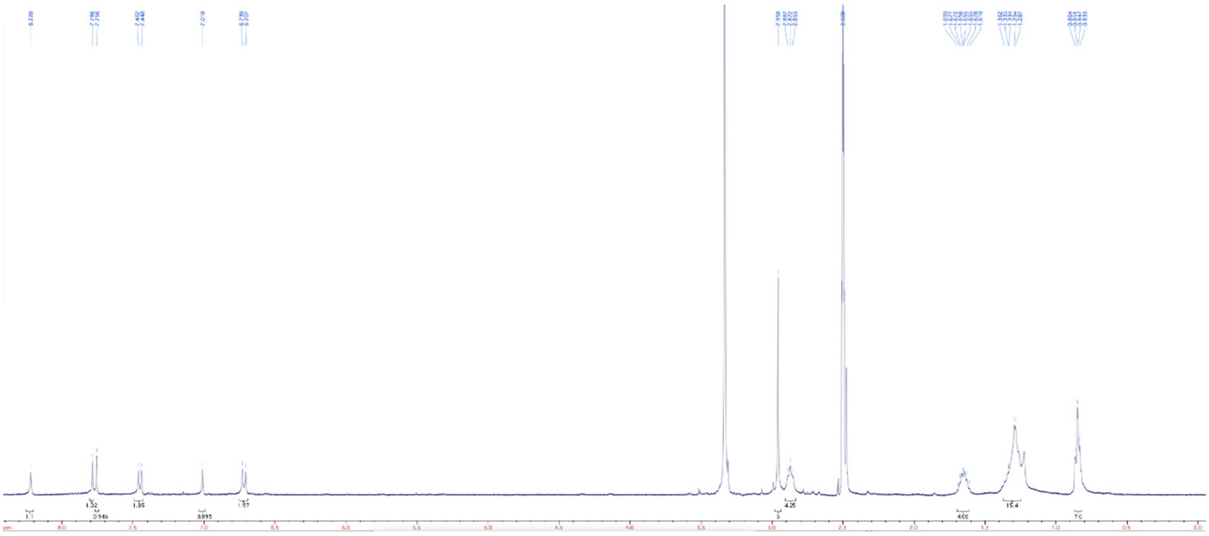

(b)

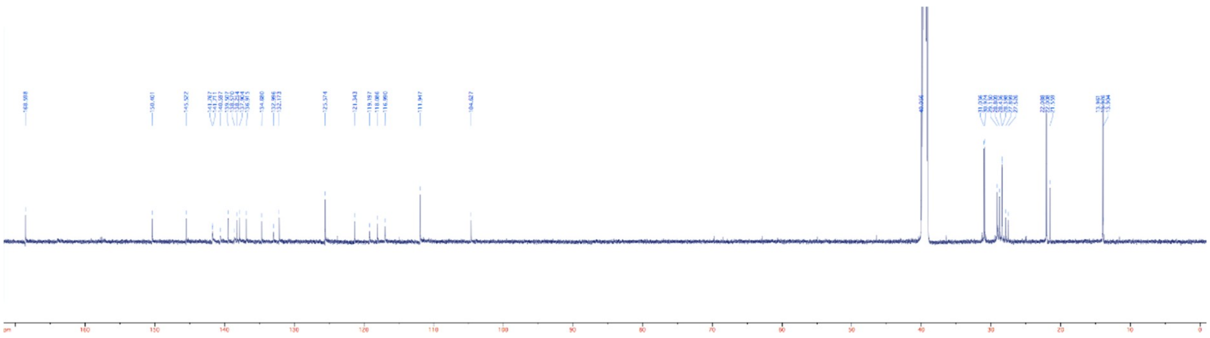

(c)

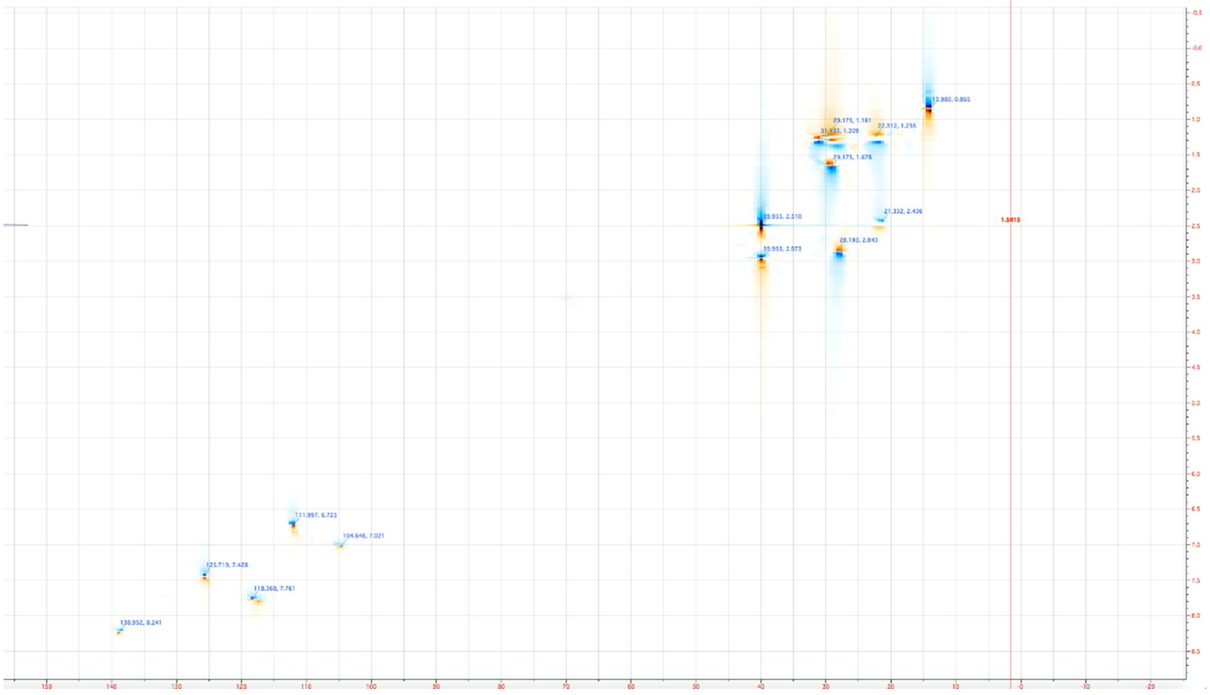

(d)

Figure S2. (a) FTIR, (b) 1H NMR, (c) ^{13}C NMR, and (d) HSQC spectra of **CSD-04.**

© 2019 by the authors. Submitted for possible open access publication under the terms and conditions of the Creative Commons Attribution (CC BY) license (http://creativecommons.org/licenses/by/4.0/).
